# Identification of multiple system atrophy mimicking Parkinson’s disease or progressive supranuclear palsy

**DOI:** 10.1093/brain/awab017

**Published:** 2021-04-05

**Authors:** Yasuo Miki, Eiki Tsushima, Sandrine C Foti, Kate M Strand, Yasmine T Asi, Adam Kenji Yamamoto, Conceição Bettencourt, Marcos C B Oliveira, Eduardo De Pablo-Fernández, Zane Jaunmuktane, Andrew J Lees, Koichi Wakabayashi, Thomas T Warner, Niall Quinn, Janice L Holton, Helen Ling

**Affiliations:** 1 Queen Square Brain Bank for Neurological Disorders, UCL Queen Square Institute of Neurology, London WC1N 1PJ, UK; 2 Department of Neuropathology, Institute of Brain Science, Hirosaki University Graduate School of Medicine, Hirosaki 036-8562, Japan; 3 Department of Comprehensive Rehabilitation Science, Hirosaki University Graduate School of Health Sciences, Hirosaki 036-8564, Japan; 4 Lysholm Department of Neuroradiology, National Hospital for Neurology and Neurosurgery, Queen Square, London, UK; 5 Department of Brain Repair and Rehabilitation, UCL Queen Square Institute of Neurology, University College London, Queen Square, London, UK; 6 Department of Neurodegenerative Disease, UCL Queen Square Institute of Neurology, University College London, London, UK; 7 Department of Neurology, Hospital das Clínicas, Faculdade de Medicina da Universidade de São Paulo (FMUSP), São Paulo, Brazil; 8 Neurology Unit, Instituto do Câncer do Estado de São Paulo (ICESP), Faculdade de Medicina da Universidade de São Paulo (FMUSP), São Paulo, Brazil; 9 Reta Lila Weston Institute of Neurological Studies, UCL Queen Square Institute of Neurology, London WC1N 1PJ, UK; 10 Department of Clinical and Movement Neurosciences, UCL Queen Square Institute of Neurology, University College London, London, UK; 11 UCL Queen Square Institute of Neurology, London WC1N 3BG, UK

**Keywords:** multiple system atrophy, Parkinson’s disease, progressive supranuclear palsy, autonomic dysfunction, red flag

## Abstract

We studied a subset of patients with autopsy-confirmed multiple system atrophy who presented a clinical picture that closely resembled either Parkinson’s disease or progressive supranuclear palsy. These mimics are not captured by the current diagnostic criteria for multiple system atrophy. Among 218 autopsy-proven multiple system atrophy cases reviewed, 177 (81.2%) were clinically diagnosed and pathologically confirmed as multiple system atrophy (i.e. typical cases), while the remaining 41 (18.8%) had received an alternative clinical diagnosis, including Parkinson’s disease (i.e. Parkinson’s disease mimics; *n = *16) and progressive supranuclear palsy (i.e. progressive supranuclear palsy mimics; *n = *17). We also reviewed the clinical records of another 105 patients with pathologically confirmed Parkinson’s disease or progressive supranuclear palsy, who had received a correct final clinical diagnosis (i.e. Parkinson’s disease, *n = *35; progressive supranuclear palsy-Richardson syndrome, *n = *35; and progressive supranuclear palsy-parkinsonism, *n = *35). We investigated 12 red flag features that would support a diagnosis of multiple system atrophy according to the current diagnostic criteria. Compared with typical multiple system atrophy, Parkinson’s disease mimics more frequently had a good levodopa response and visual hallucinations. Vertical gaze palsy and apraxia of eyelid opening were more commonly observed in progressive supranuclear palsy mimics. Multiple logistic regression analysis revealed an increased likelihood of having multiple system atrophy [Parkinson’s disease mimic versus typical Parkinson’s disease, odds ratio (OR): 8.1; progressive supranuclear palsy mimic versus typical progressive supranuclear palsy, OR: 2.3] if a patient developed any one of seven selected red flag features in the first 10 years of disease. Severe autonomic dysfunction (orthostatic hypotension and/or urinary incontinence with the need for a urinary catheter) was more frequent in clinically atypical multiple system atrophy than other parkinsonian disorders (Parkinson’s disease mimic versus typical Parkinson’s disease, OR: 4.1; progressive supranuclear palsy mimic versus typical progressive supranuclear palsy, OR: 8.8). The atypical multiple system atrophy cases more frequently had autonomic dysfunction within 3 years of symptom onset than the pathologically confirmed patients with Parkinson’s disease or progressive supranuclear palsy (Parkinson’s disease mimic versus typical Parkinson’s disease, OR: 4.7; progressive supranuclear palsy mimic versus typical progressive supranuclear palsy, OR: 2.7). Using all included clinical features and 21 early clinical features within 3 years of symptom onset, we developed decision tree algorithms with combinations of clinical pointers to differentiate clinically atypical cases of multiple system atrophy from Parkinson’s disease or progressive supranuclear palsy.

## Teaser

A subset of patients with multiple system atrophy (MSA) present with a clinical picture that closely resembles either Parkinson’s disease or progressive supranuclear palsy (PSP). By studying autopsy-confirmed cases, Miki *et al.* provide clinical pointers that can help identify MSA mimicking Parkinson’s disease or PSP.

See Di Luca and Lang (doi.10.1093/brain/awab115) for a scientific commentary on this article.

## Introduction

Multiple system atrophy (MSA) is a sporadic neurodegenerative disease characterized by the presence of autonomic failure, levodopa unresponsive parkinsonism, and/or cerebellar ataxia.^1–4^ Neuropathologically, abnormal α-synuclein accumulates in oligodendrocytes and, to a lesser extent, neurons in association with neurodegeneration in the striatonigral or olivopontocerebellar structures.^5^^-^^7^ The current diagnostic criteria define two clinical types of MSA: one with predominant parkinsonism (MSA-P), reflecting striatonigral degeneration (SND), and one with predominant cerebellar ataxia (MSA-C) related to olivopontocerebellar atrophy (OPCA). The current operational diagnostic criteria allow for three degrees of clinical diagnostic certainty: ‘definite’, ‘probable’ and ‘possible’ MSA. Neuropathological confirmation is required for a ‘definite’ diagnosis.^8^

The diagnosis of atypical parkinsonian syndromes including MSA is often difficult, particularly in the early years of disease, and errors of both omission and commission are common. Up to 50% of patients with MSA experience a significant beneficial but not usually sustained response to levodopa, which argues against a diagnosis of ‘probable’ MSA.^8–10^ Patients with autopsy-proven Parkinson’s disease can show varying degrees of autonomic dysfunction and the response to levodopa, particularly in the elderly, may not be striking.^11^^,^^12^ In an earlier study, we investigated 203 patients with a final clinical diagnosis of MSA, 43 (21%) some of whom had different pathological diagnoses, mainly Parkinson’s disease (*n = *26) or progressive supranuclear palsy (PSP) (*n = *13). All misdiagnosed cases had autonomic dysfunction plus other features supportive of a clinical diagnosis of MSA that made them ‘atypical’ enough for a diagnosis of MSA to have been justified on clinical grounds.^12^ It is also recognized that a proportion of patients with MSA develop clinical features suggestive of Parkinson’s disease or PSP. Red flag features, defined as clinical features to support a diagnosis of MSA, occur less frequently in clinically atypical MSA.^8^^,^^13^ In fact, previous clinicopathological studies have found that 12–29% and 6% of autopsy-proven MSA cases had an antemortem diagnosis of Parkinson’s disease (Parkinson’s disease mimics) and PSP (PSP mimics), respectively.^14–16^ Thus, atypical MSA cases, who would not have met the current diagnostic criteria for MSA, are likely to be erroneously diagnosed as having Parkinson’s disease or PSP in life. However, previous clinicopathological studies have not systematically studied these cases as separate subgroups so that much of the existing clinical literature consists of the clinical features of a mixture of both typical and atypical MSA cases.

In the present study, we investigated 249 consecutive patients with autopsy-proven MSA and divided them into several clinical subtypes including typical MSA, Parkinson’s disease mimics and PSP mimics. To characterize the atypical clinical features of the Parkinson’s disease mimics and the PSP mimics, we compared them with those observed in patients with clinically and pathologically diagnosed Parkinson’s disease (typical Parkinson’s disease: *n = *35) or PSP [typical PSP: PSP-Richardson syndrome (PSP-RS), *n = *35; PSP-parkinsonism (PSP-P): *n = *35].

## Materials and methods

### Patients

In total, 354 autopsy-confirmed cases were investigated. These included 249 consecutive patients with a neuropathological diagnosis of MSA referred to the Queen Square Brain Bank (QSBB) for Neurological Disorders between 1989 and 2018, and patients with clinically and pathologically confirmed Parkinson’s disease (*n = *35) or PSP (PSP-RS: *n = *35; PSP-P: *n = *35). From the QSBB database, the most recent 35 consecutive pathologically confirmed Parkinson’s disease and 35 pathologically confirmed PSP cases fulfilling the corresponding clinical diagnostic criteria were selected: clinically established Parkinson’s disease (*n = *26/35), clinically probable Parkinson’s disease (*n = *9/35), probable PSP-RS (*n = *35/35) and probable PSP-P (*n = *30/35), suggestive PSP-P (*n = *5/35).^17–19^ The clinical features of 176 of these 249 autopsy-confirmed MSA cases have been previously reported.^12^

### Medical record review

**Figure 1:**
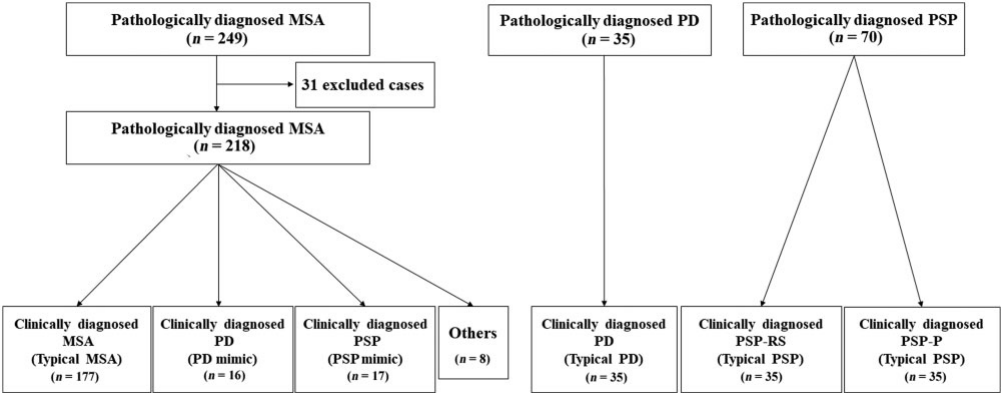
**Study design** **of the present study.**

All available medical records of the 354 cases were systematically reviewed. This included the primary care medical records, correspondence between medical specialists and general practitioners and the medical files from the National Hospital for Neurology and Neurosurgery Queen Square, London. All patients had been assessed by at least one hospital specialist (consultant physician, geriatrician or neurologist) during the course of their illness. Ninety-eight per cent (213/218) of patients had been reviewed at least once by a neurologist. Thirty-one cases were excluded from the 249 MSA cases because of inadequate medical records relating to the course of the disease (*n = *23), severe autonomic neuropathy related to other causes including diabetic autonomic neuropathy (*n = *6), deep brain stimulation (*n = *1) and severe post-mortem artefact precluding reliable pathological evaluation (*n = *1) (Fig. 1).

We systematically reviewed the clinical features that were either supportive or non-supportive of a diagnosis of ‘probable’ and ‘possible’ MSA as outlined in the current diagnostic criteria for MSA.^8^ To characterize atypical MSA cases masquerading as PSP, core features of PSP-RS or PSP-P cases were also systematically reviewed. All clinical features are listed in the Supplementary material. All clinical signs or symptoms were recorded as unknown if they were not specifically mentioned.

### Neuropathological methods

All included cases were part of the archival collection of the QSBB donation program, which follows ethically approved protocols, and the tissues were stored under a licence from the Human Tissue Authority. The brains were fixed in 10% buffered formalin for 3 weeks. Immunohistochemical analysis was performed using 8-μm thick, formalin-fixed, paraffin-embedded sections from multiple brain regions. The sections were subjected to immunohistochemical processing using the avidin-biotin-peroxidase complex method with diaminobenzidine as the chromogen. The primary antibodies used were anti-amyloid-β (M0872; Dako; 1:100), anti-α-synuclein (MA1-90342; Thermo Scientific; 1:1500) and anti-phosphorylated tau (MN1020; Thermo Scientific; 1:600) antibodies. The Bielschowsky silver impregnation was performed in some cases for the assessments of neuritic plaques and neurofibrillary tangles. MSA was classified into four pathological subtypes: MSA, striatonigral degeneration predominant type (MSA-SND); MSA, olivopontocerebellar predominant type (MSA-OPCA); MSA with equal involvement of SND and OPCA (MSA-SND=OPCA); and MSA with minimal change based on previously published criteria.^20^^,^^21^ Lewy pathology [brainstem-predominant, limbic (transitional) and diffuse neocortical] was assigned based upon the pattern of Lewy body-related pathology according to the consensus criteria for pathological assessment of dementia with Lewy bodies.^22^ We used sections stained with haematoxylin and eosin to assess concomitant Lewy bodies in the substantia nigra and the locus coeruleus due to difficulties in discriminating concomitant Lewy bodies in MSA from neuronal cytoplasmic inclusions, another characteristic feature of MSA, with the use of α-synuclein immunohistochemistry.^6^ Neuritic plaques and neurofibrillary tangles were evaluated according to the Consortium to Establish a Registry for Alzheimer’s Disease (CERAD) scheme and Braak and Braak neurofibrillary tangle stage, respectively.^23^ Amyloid-β deposits are first encountered in the basal portions of the frontal, temporal and occipital lobes, and then spread to other regions.^24^ If the parietal lobe was not available for evaluating neuritic plaques, we evaluated neuritic plaques using the frontal and temporal lobes that are considered to have more amyloid deposits than the parietal lobe.

### Statistical analysis

All statistical analyses in the present study were performed using SPSS 25.0 (SPSS Inc., USA) or R commander. The chi-square or Fisher’s exact tests with Bonferroni correction was used to compare categorical variables. We performed one-way ANOVA followed by Tukey test for parametric data or Kruskal-Wallis test followed by Steel-Dwass test for non-parametric data. We also performed multiple logistic regression analysis to estimate the association among clinical subtypes in CERAD plaque scheme, Braak and Braak neurofibrillary tangle stage, autonomic dysfunction score or red flag score. As accumulation of tau and amyloid-β is influenced by the ageing process,^25^ we adjusted for age in these evaluations. Odds ratio (OR) and 95% confidence interval (CI) were used as the main effect size. To help differentiate between clinically atypical MSA and typical Parkinson’s disease or PSP, we performed decision tree analysis (chi-square automatic interaction detection) using all available clinical features. A probability value of less than 0.05 (*P < *0.05) was considered to be significant.

### Data availability

The data are not publicly available due to the inclusion of information that could compromise the privacy of research participants.

## Results

### Demographic data

Table 1 shows the demographic data of all patients in the present study. Of 218 patients with a pathological diagnosis of MSA, 177 (81.2%) received a clinical diagnosis of MSA (typical MSA). The remaining 41 (18.8%) patients had a different clinical diagnosis at death: Parkinson’s disease (Parkinson’s disease mimics: *n = *16) and PSP (PSP mimics: *n = *17). The clinical diagnoses for the remaining eight cases were: corticobasal syndrome (*n = *2), vascular parkinsonism (*n = *2), ‘atypical parkinsonism of uncertain cause’ (*n = *2), pure autonomic failure (*n = *1) and undiagnosed (*n = *1). Of the remaining atypical MSA cases, 87.5% (14/16) of Parkinson’s disease mimics, 100% (17/17) of PSP mimics, and 100% (8/8) were reviewed and followed up by experienced neurologists. Retrospective assessment of clinical features showed that 83 (46.9%) and 71 (40.1%) of typical MSA cases met the criteria-defined diagnosis for ‘probable’ and ‘possible’ MSA, respectively. Twenty-three (13%) typical MSA cases lacked aspects of the clinical information required for the current diagnostic criteria. Of 16 Parkinson’s disease mimics, one fulfilled a diagnosis of ‘clinically established’ Parkinson’s disease and eight had a diagnosis of ‘clinically probable’ Parkinson’s disease.^19^ Eight patients fulfilled the QSBB Criteria for the diagnosis of Parkinson’s disease.^26^ Of 17 PSP mimics, six fulfilled a diagnosis of ‘probable PSP-RS or PSP-P’, two a diagnosis of ‘possible PSP with progressive gait freezing’ and three a diagnosis of ‘suggestive of PSP-P’.^17^^,^^18^ Clinically, PSP mimics had older age at onset than typical MSA cases (typical MSA versus PSP mimics: 56.0 ± 9.0 versus 61.7 ± 5.4, *P < *0.05). Parkinson’s disease mimics had a shorter time to final diagnosis than typical MSA or PSP mimics (typical MSA versus Parkinson’s disease mimics, 4.2 ± 2.5 versus 1.3 ± 1.5, *P < *0.01; PSP mimics versus Parkinson’s disease mimics, 3.9 ± 1.7 versus 1.3 ± 1.5, *P < *0.01). For those patients who eventually received a final clinical diagnosis of MSA-P (*n = *110), 72 had an initial diagnosis of Parkinson’s disease, which was then revised to MSA when core MSA features such as autonomic dysfunction or ataxia emerged. Such revision of clinical diagnosis did not happen in Parkinson’s disease mimics due to the absence of autonomic dysfunction and/or ataxia, or these features not being severe enough to meet the diagnostic criteria for MSA. There were no significant differences in the proportion of male/female, age at death, disease duration to death, and latency between last examination and death between typical MSA, Parkinson’s disease mimics and PSP mimics.

**Table 1:** Demographic data

Pathological diagnosis	MSA				PD	PSP	
	Typical MSA	PD mimic	PSP mimic	Others^a^	Typical PD	Typical PSP (PSP-RS)	Typical PSP (PSP-P)
**Clinical features**							
Number of patients (%)	177 (81.2)	16 (7.3)	17 (7.8)	8 (3.7)	35	35	35
Male, % (*n*)	54.8 (97/177)	50 (8/16)	23.5 (4/17)	37.5 (3/8)	45.7 (16/35)	57.1 (20/35)	80 (28/35)
Age at onset, years, mean ± SD	56.0 ± 9.0	61.9 ± 10.7	61.7 ± 5.4	56.4 ± 8.5	56.1 ± 11.7	66.1 ± 7.9	62.6 ± 10.4
Age at death, years, mean ± SD	63.6 ± 8.2	68.7 ± 9.5	67.5 ± 5.4	62.5 ± 9.3	78.2 ± 8.1	74.3 ± 7.2	73.1 ± 10.7
Time to final diagnosis, years, mean ± SD	4.2 ± 2.5	1.3 ± 1.5	3.9 ± 1.7	4.1 ± 2.3	1.7 ± 1.8	3.6 ± 2.2	6.2 ± 2.6
Disease duration to death, years, mean ± SD	7.6 ± 3.0	7.1 ± 4.1	6.4 ± 1.8	6.1 ± 2.4	21.9 ± 6.6	8.0 ± 3.0	10.5 ± 4.0
Latency between last examination and death, years, mean ± SD	1.1 ± 1.1	0.8 ± 0.9	0.8 ± 0.9	0.9 ± 1.0	2.1 ± 2.7	1.2 ± 1.6	1.4 ± 1.7
MSA clinical subtypes							
MSA-P, % (*n*)	62.1 (110/177)	NA	NA	NA	NA	NA	NA
MSA-C, % (*n*)	37.9 (67/177)	NA	NA	NA	NA	NA	NA
**Pathological features**							
MSA pathological subtype							
MSA-SND, % (*n*)	29.4 (52/177)	50 (8/16)	70.6 (12/17)	50 (4/8)	NA	NA	NA
MSA-OPCA, % (*n*)	34.5 (61/177)	18.8 (3/16)	0 (0/17)	12.5 (1/8)	NA	NA	NA
MSA-mixed, % (*n*)	33.9 (61/177)	31.3 (5/16)	29.4 (5/17)	12.5 (1/8)	NA	NA	NA
Minimal change, % (*n*)	1.7 (3/177)	0 (0/16)	0 (0/17)	25 (2/8)	–	–	–
CERAD plaque score, median (25th, 75th percentile)	0 (0, 0)	0 (0, 3)	0 (0, 1)	0 (0,0)	0 (0, 1)	0 (0, 1)	0 (0,1)
NFT stage, median (25th, 75th percentile)	I (0, I)	0 (0, 0)	I (0.5, I)	0 (0,0)	II (I, II)	II (0, II)	II (0, II)
Lewy body pathology, % (*n*)	7.3 (13/177)	12.5 (2/16)	5.9 (1/17)	0 (0/8)	100 (35/35)	22.9 (8/35)	17.1 (6/35)
Brainstem-predominant, % (*n*)	NA	NA	NA	NA	0 (0/35)	NA	NA
Limbic (transitional), % (*n*)	NA	NA	NA	NA	14.3 (5/35)	NA	NA
Diffuse neocortical, % (*n*)	NA	NA	NA	NA	85.7 (30/35)	NA	NA

CERAD = the Consortium to Establish a Registry for Alzheimer’s Disease; NA = not applicable; NFT = neurofibrillary tangles; PD = Parkinson’s disease; SD = standard deviation.

a Autopsy-confirmed MSA cases with other final clinical diagnoses: corticobasal syndrome (*n = *2), vascular parkinsonism (*n = *2), atypical parkinsonism of uncertain cause (*n = *2), pure autonomic failure (*n = *1) and undiagnosed (*n = *1).

When comparing pathological subtypes,^21^ PSP mimics had a higher proportion of MSA-SND (typical MSA versus PSP mimics: 29.4% versus 70.6%, *P < *0.01) and lower proportion of MSA-OPCA (34.5% versus 0%, *P < *0.05) than typical MSA cases. We also examined whether the proportion of MSA-SND or -OPCA differed between MSA-P cases and PSP mimics, but found no difference. There were five cases with ‘minimal change MSA’ in the present study.^20^ Three of five received a clinical final diagnosis of MSA and the remaining two received a clinical diagnosis of atypical parkinsonism or pure autonomic failure. Compared to typical MSA cases, Parkinson’s disease mimics had higher CERAD plaque scores (*P < *0.01) and lower neurofibrillary tangle stages (*P < *0.05) after adjusting for age. No correlation was found between the occurrence of visual hallucinations and concomitant Lewy bodies in patients with pathologically confirmed MSA (data not shown). Additional demographic data of Parkinson’s disease or PSP mimics are shown in Supplementary Table 1.

### Characteristics of atypical MSA: Parkinson’s disease mimics

#### Case illustration

A 67-year-old female (Case 11 in Supplementary Table 2): presented with a 1-year history of worsening dexterity of her left hand. Examination revealed an asymmetrical tremor of the hand at rest with cogwheel rigidity and bradykinesia. Her symptoms and signs improved with levodopa therapy (62.5 mg four times a day). Over the next 3 years, she developed motor fluctuations, generalized peak-dose dyskinesia, worsening dysarthria, urinary frequency and constipation. She also reported intermittent mild non-threatening visual hallucinations. Orofacial dystonia and urinary incontinence then became more intrusive. She died aged 75. The final clinical diagnosis was Parkinson’s disease. Autopsy confirmed the pathological diagnosis of MSA (SND=OPCA subtype). There were frequent depositions of neuritic plaques (CERAD C3) but no neurofibrillary tangles were found in the brain. Key clinical features of all Parkinson’s disease mimics (Cases 1–16) are provided in Supplementary Table 2.

#### Atypical MSA (Parkinson’s disease mimic) versus typical MSA or Parkinson’s disease

To characterize the clinical features of Parkinson’s disease mimics (*n = *16), we compared all key clinical features including motor symptoms, levodopa response, depression, visual hallucinations, RBD (Supplementary Table 3) with those in typical MSA (*n = *177) and typical Parkinson’s disease (*n = *35). The frequencies of these features over the course are shown in Supplementary Fig. 1. Typical Parkinson’s disease cases had a longer mean disease duration (21.9 years) than typical MSA cases or Parkinson’s disease mimics (7.6 or 7.1 years, respectively). Of the 218 MSA cases in this study, 48 (22%) had a disease duration between 10 and 18 years. In addition, our group reported four MSA cases with disease duration of 15 years or more. Initially, their clinical presentation resembled Parkinson’s disease with sustained levodopa response. Autonomic dysfunction only emerged a decade later, revealing the underlying diagnosis of MSA.^27^ Thus, in Supplementary Fig. 1, we investigated the frequencies of 10 features within 10 years of symptom onset, which were significantly different in the first 3 years or during lifetime between the three groups. More Parkinson’s disease mimics had a sustained levodopa response (typical MSA versus Parkinson’s disease mimic; 41.1% versus 81.3%; *P < *0.01), early rigidity within 3 years of onset (32.8% versus 68.8%; *P* < 0.05), visual hallucinations withing 10 years of onset (4% versus 25%; *P < *0.05) and visual hallucinations (5.1% versus 25%; *P < *0.05) than typical MSA patients. 69.2% (9/13) of Parkinson’s disease mimics with a sustained levodopa response developed either dyskinesia, ON/OFF phenomena or wearing off. None of the Parkinson’s disease mimics developed symptoms such as postural/action tremor (33.3% versus 0%, *P < *0.05), ataxia (64.4% versus 0%, *P < *0.01) and stridor (31.1% versus 0%; *P < *0.05) that would have led the clinicians to question their clinical diagnosis of Parkinson’s disease. REM sleep behaviour disorder (RBD) was also less frequently observed in Parkinson’s disease mimics compared with typical MSA cases (41.8% versus 6.3%; *P < *0.05) (Supplementary Table 3). When compared between Parkinson’s disease mimic and typical Parkinson’s disease, the frequencies of the examined clinical did not differ significantly except for resting tremor within 10 years of onset (Parkinson’s disease mimic versus typical Parkinson’s disease; 25% versus 80%; *P < *0.01), resting tremor (25% versus 91.4%; *P < *0.01), falls within 10 years of onset (87.5% versus 31.4%; *P < *0.01), freezing of gait (25% versus 68.6%; *P < *0.05) and visual hallucination (25% versus 80%; *P < *0.01) (Supplementary Table 3 and Supplementary Fig. 1). None of the typical MSA, Parkinson’s disease mimic and typical Parkinson’s disease cases developed early apraxia of eyelid opening within 3 years of onset (data not shown).

A number of clinical features, also described as red flag features, have previously been used to differentiate MSA from Parkinson’s disease.^13^ Among them, in the present study, typical MSA patients were more frequently recorded to have inspiratory sighs (typical MSA versus typical Parkinson’s disease: 20.3% versus 2.9%; *P < *0.05), jerky myoclonic postural/action tremor (23.7% versus 5.7%; *P < *0.05), polyminimyoclonus (14.7% versus 0%; *P < *0.05), severe dysarthria (46.9% versus 5.7%; *P < *0.01) and snoring (28.2% versus 2.9%; *P < *0.05) throughout the disease course when compared with typical Parkinson’s disease. In addition, snoring was more common in typical MSA cases than in Parkinson’s disease mimics (*P < *0.05). There was no difference in frequencies of these clinical features between Parkinson’s disease mimics and typical Parkinson’s disease (Table 2).

**Table 2:** Red flag features in atypical MSA (Parkinson’s disease mimic) versus typical MSA or Parkinson’s disease

Pathological diagnosis	MSA		Parkinson's disease
	Typical MSA (*n *=* *177)	Parkinson’s disease mimic (*n *=* *16)	Typical Parkinson’s disease (*n *=* *35)
**The frequency of red flag features during lifetime**			
1. Orofacial dystonia, % (*n*)	11.9 (21/177)	6.3 (1/16)	8.6 (3/35)
2. Inspiratory sighs, % (*n*)	20.3 (36/177)**	6.3 (1/16)	2.9 (1/35)
3. Contractures of hands and feet, % (*n*)	9.0 (16/177)	12.5 (2/16)	2.9 (1/35)
4. Jerky myoclonic postural/action tremor, % (*n*)	23.7 (42/177)**	0 (0/16)	5.7 (2/35)
5. Polyminimyoclonus, % (*n*)	14.7 (26/177)**	0 (0/16)	0 (0/35)
6. Severe dysphonia, % (*n*)	22.6 (40/177)	12.5 (2/16)	8.6 (3/35)
7. Severe dysarthria, % (*n*)	46.9 (83/177)***	25 (4/16)	5.7 (2/35)
8. Pathological laughter or crying, % (*n*)	23.2 (41/177)	0 (0/16)	5.7 (2/35)
9. Snoring, % (*n*)	28.2 (50/177)*^,^***	0 (0/16)	2.9 (1/35)
10. Disproportionate antecollis, % (*n*)	16.4 (29/177)	12.5 (2/16)	2.9 (1/35)
11. Camptocormia and/or Pisa syndrome, % (*n*)	2.8 (5/177)	12.5 (2/16)	8.6 (3/35)
12. Cold hands and feet, % (*n*)	20.3 (36/177)	6.3 (1/16)	5.7 (2/35)
**The number of red flag features**			
1. Cumulative frequency during lifetime			
Red flag score (the combination of red flags: 1, 2, 3, 5, 7, 8, 12), mean ± SD (OR, 95% CI)	1.5 ± 1.2 (4.6, 2.4–8.6)***	0.6 ± 0.6	0.3 ± 0.6
2. Cumulative frequency within 10 years of onset			
Red flag score (the combination of red flags: 1, 2, 3, 5, 7, 8, 12), mean ± SD (OR, 95% CI)	1.3 ± 1.1 (25.1, 6.0–105.5)***	0.4 ± 0.6 (8.1, 1.6–39.6)^†^	0.1 ± 0.2
3. Cumulative frequency within 3 years of onset			
Red flag score (1–12), mean ± SD (OR, 95% CI)	0.4 ± 0.8 (NA)	0.3 ± 0.4 (NA)	0

NA = not applicable.

* *P *<* *0.05 typical MSA versus Parkinson’s disease mimic.

** *P *<* *0.05 typical MSA versus typical Parkinson’s disease.

*** *P *<* *0.01 typical MSA versus typical Parkinson’s disease.

† *P *<* *0.05 PD mimic versus typical Parkinson’s disease.

Previously, we identified seven selected features to differentiate MSA from autopsy-proven Parkinson’s disease cases that masqueraded as MSA in life (MSA mimics)^12^: orofacial dystonia, inspiratory sighs, contractures of hands and feet, polyminimyoclonus, severe dysarthria, pathological laughter or crying, and cold hands and feet (Table 2). In the present study, we performed multiple logistic regression analysis to address whether the same combination of features were useful in discriminating atypical MSA (Parkinson’s disease mimic) from typical Parkinson’s disease. While typical MSA patients accumulated more of these features than typical Parkinson’s disease patients (OR: 4.6, 95% CI: 2.4–8.6, *P < *0.01), we found no difference in frequencies of the seven red flag features between Parkinson’s disease mimic and typical Parkinson’s disease. In clinical practice, many typical Parkinson’s disease cases can develop some of these features in the later stages of their disease. In addition, in the current diagnostic criteria for Parkinson’s disease, the presence of disproportionate antecollis or contractures of hands or feet within the first 10 years are considered features that do not support a diagnosis of Parkinson’s disease.^19^ Thus, we focused our analysis on the first 10 years after disease onset. When compared with typical Parkinson’s disease cases, the frequencies of these features within 10 years of disease onset was significantly higher in typical MSA (typical MSA versus typical Parkinson’s disease, OR: 25.1, 95% CI: 6.0–105.5, *P < *0.01) and Parkinson’s disease mimics (Parkinson’s disease mimics versus typical Parkinson’s disease, OR: 8.1, 95% CI: 1.6–39.6, *P < *0.05). These findings suggest an increased likelihood of having MSA if a patient develops one of the seven selected features within 10 years of disease onset, with or without symptoms indicative of Parkinson’s disease including beneficial levodopa response or visual hallucinations. Notably, none of the seven red flag features was observed in any typical Parkinson’s disease patient within 3 years of disease onset. Multiple logistic regression analysis using typical Parkinson’s disease as a reference was therefore not performed.

We then investigated whether the frequency of autonomic dysfunction differed between typical MSA, Parkinson’s disease mimic and typical Parkinson’s disease (Table 3 and Supplementary Fig. 2). Compared to typical Parkinson’s disease, Parkinson’s disease mimics more often developed autonomic dysfunctions including urinary urgency, frequency, incomplete bladder emptying, or mild orthostatic hypotension within 10 years of onset, and urinary incontinence within 3 and 10 years of onset (Supplementary Fig. 2A and B). However, there was no difference in severe orthostatic hypotension throughout the disease course between Parkinson’s disease mimic and typical Parkinson’s disease. The low frequency of severe orthostatic hypotension may have contributed to the misdiagnosis of Parkinson’s disease in these atypical MSA cases. In our previous study, we found the presence of a combination of autonomic dysfunction (severe orthostatic hypotension and/or urinary incontinence with use of urinary catheters) useful to rule out atypical Parkinson’s disease patients, who masqueraded as MSA in life (MSA mimics), from patients with MSA.^12^ Multiple logistic analysis using the same combination revealed an increased likelihood of having MSA (typical MSA versus typical Parkinson’s disease, OR: 17.1, 95% CI: 6.5–45.2, *P < *0.01; Parkinson’s disease mimic versus typical Parkinson’s disease, OR: 4.1, 95% CI: 1.2–13.6, *P < *0.05). We further examined the number of features of autonomic dysfunction within 3 years of disease onset: urinary urgency, frequency, incontinence, incomplete bladder emptying, or mild or severe orthostatic hypotension. Both typical MSA patients and Parkinson’s disease mimics more frequently had autonomic dysfunction in an early disease stage within 3 years of onset than typical Parkinson’s disease patients (typical MSA versus typical Parkinson’s disease, OR: 7.0, 95% CI: 2.7–18.5, *P < *0.01; Parkinson’s disease mimic versus typical Parkinson’s disease, OR: 4.7, 95% CI: 1.6–14.0, *P < *0.01). Thus, despite having clinical features suggestive of Parkinson’s disease, patients with MSA usually develop earlier and more severe autonomic dysfunction than patients with Parkinson’s disease.

**Table 3:** Autonomic dysfunction in atypical MSA (Parkinson’s disease mimic) versus typical MSA or Parkinson’s disease

Pathological diagnosis	MSA		Parkinson’s disease
	Typical MSA (*n *=* *177)	Parkinson’s disease mimic (*n *=* *16)	Typical Parkinson’s disease (*n *=* *35)
**The frequency of autonomic dysfunction**			
Autonomic dysfunction (any including constipation), % (*n*)	100 (177/177)***	93.8 (15/16)	85.7 (30/35)
Urinary urgency, frequency, incomplete bladder emptying, or mild orthostatic hypotension, % (*n*)	76.3 (135/177)	75 (12/16)	57.1 (20/35)
Early urinary urgency, frequency, incomplete bladder emptying, or mild orthostatic hypotension, % (*n*) within 3 years of onset	41.8 (74/177)***	31.3 (5/16)	5.7 (2/35)
Urinary incontinence, % (*n*)	76.3 (135/177)***	68.8 (11/16)	45.7 (16/35)
Early urinary incontinence within 3 years of onset, % (*n*)	31.1 (55/177)^***^	31.3 (5/16)^†^	2.9 (1/35)
Severe orthostatic hypotension, % (*n*)	58.8 (104/177)*^,^***	18.8 (3/16)	5.7 (2/35)
Early severe orthostatic hypotension within 3 years of onset, % (*n*)	18.1 (32/177)**	0 (0/16)	0 (0/35)
**The number of autonomic dysfunction during lifetime**			
Severe orthostatic hypotension and/or urinary incontinence with use of urinary catheters, mean ± SD (OR, 95% CI)	1.2 ± 0.7 (17.1, 6.5–45.2)***	0.5 ± 0.7 (4.1, 1.2–13.6)^†^	0.1 ± 0.4
**The number of autonomic dysfunction within 3 years of onset**			
Urinary urgency, frequency, incontinence, incomplete bladder emptying, or mild or severe orthostatic hypotension, mean ± SD (OR, 95% CI)	1.1 ± 1.1 (7.0, 2.7–18.5)***	0.7 ± 0.9 (4.7, 1.6–14.0)^††^	0.1 ± 0.5

* *P *<* *0.01 typical MSA versus Parkinson’s disease mimic.

** *P *<* *0.05 typical MSA versus typical Parkinson’s disease.

*** *P *<* *0.01 typical MSA versus typical Parkinson’s disease.

† *P *<* *0.05 PD mimic versus typical Parkinson’s disease.

†† *P *<* *0.01 PD mimic versus typical Parkinson’s disease.

**Figure 2:**
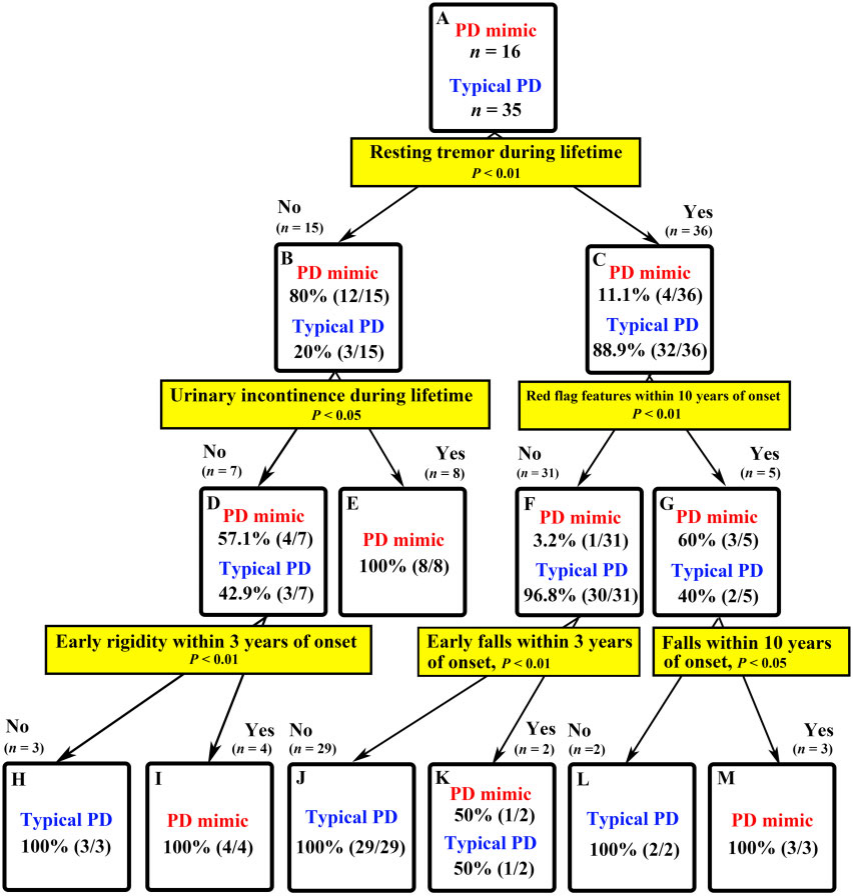
**Decision tree analysis: Parkinson’s disease mimic versus typical Parkinson’s disease.** Using all clinical features examined (75 items) outlined in Tables 2 and 3, Supplementary Table 3, and Supplementary Figs 1 and 2, decision tree analysis was performed to distinguish Parkinson’s disease mimics from typical Parkinson’s disease. The decision tree algorithm systemically selects the most determinant feature in the diagnostic process for the present cohort. It suggests which clinical feature should be heeded at the time of examination and/or in the future. Percentages indicate the probability of the underlying pathology [atypical MSA (Parkinson’s disease mimics) or typical Parkinson’s disease].

Finally, using 75 clinical features investigated in the present study, we performed decision tree analysis to identify Parkinson’s disease mimics among patients with an antemortem diagnosis of Parkinson’s disease (Fig. 2). These 75 items are outlined in Table 2 (15 items of red flag features), Table 3 (nine items of autonomic dysfunction), Supplementary Table 3 (38 items of clinical features), Supplementary Figs 1 and 2 (13 clinical features within 10 years of onset). The decision tree algorithm systemically selects the most determinant feature in the diagnostic process for this cohort. Thus, this algorithm depicts possible outcomes by examining the presence of discriminators when making a clinical differential diagnosis between Parkinson’s disease mimic and typical Parkinson’s disease. In patients developing symptoms suggestive of both MSA and Parkinson’s disease, the presence of resting tremor is first to be checked (Fig. 2A). Then, clinical symptoms at lower levels of the algorithm should be heeded depending on results (Fig. 2B and C). At each step, the results are accompanied by percentages showing the probabilities of having either an underlying pathology of MSA or Parkinson’s disease (Fig. 2B–M). We further performed decision tree analysis using 21 early clinical features within 3 years of onset (one item in Table 2; four items in Table 3; and 16 items in Supplementary Table 3). However, because of a paucity of recorded features in early disease, we could not generate any significant results to identify Parkinson’s disease mimics at the early stage of illness (data not shown).

### Characteristics of atypical MSA: PSP mimic

#### Case illustration

A 54-year old female (Case 21 in Supplementary Table 2) developed dragging of her left leg and frequent falls. She also complained of urinary frequency and urgency that progressed to incontinence. She was initially diagnosed as having Parkinson’s disease. She then developed double vision on looking up and down at age 58, and a vertical supranuclear gaze palsy and apraxia of eyelid opening was observed at age 60. The clinical diagnosis was revised to PSP. Throughout the course of her illness, she was reported as having antecollis, contractures of her feet and a jerky myoclonic postural and action tremor of the hands. Emotional lability and pathological laughter were also prominent features in her last 3 years of life. She died 9 years after onset of her first symptoms. At autopsy, a pathological diagnosis of MSA (SND subtype) was made. Tau immunohistochemistry showed moderate numbers of neurofibrillary tangles and neuropil threads in the transentorhinal cortex (Braak and Braak stage I). There was no amyloid-β pathology in the brain. Key clinical features of all PSP mimics (Cases 17–33) are described in Supplementary Table 2.

#### Atypical MSA (PSP mimic) versus typical MSA or PSP

To characterize the clinical features of PSP mimics, we compared them between typical MSA, PSP mimics, PSP-RS and PSP-P (Supplementary Table 4). Compared with typical MSA, PSP mimics more often developed early bradykinesia within 3 years of onset (typical MSA versus PSP mimic; 46.3% versus 82.4%, *P < *0.05), positive pull test (32.2% versus 64.7%, *P < *0.05), vertical gaze palsy (up or down gaze) (20.3% versus 76.5%, *P < *0.01), apraxia of eyelid opening (4.0% versus 29.4%, *P < *0.01) and visual hallucination (5.1% versus 35.3%, *P < *0.01), and less ataxia (64.4% versus 17.6%, *P < *0.01) and stridor (31.1% versus 0%, *P < *0.01). Of the PSP mimics with vertical gaze palsy, 46.2% (6/13) developed down gaze palsy, and 38.5% (5/13) developed slow velocity of vertical saccades. On the other hand, only eight (4.5%) or four (2.3%) of 177 cases of typical MSA was reported to have down gaze palsy or slow velocity of vertical saccades in life. Visual hallucination was more frequent in PSP mimics than in cases of typical MSA even after adjustments for significantly different confounders including age at onset, the proportions of MSA-SND and MSA-OPCA between the two groups. Visual hallucinations are common in Parkinson’s disease, but are rarely seen in typical MSA and PSP.^12^^,^^28^ In these cases, the reported visual hallucinations were mild and non-threatening and not associated with delirium. There was no difference in the frequencies of clinical features between PSP mimics, PSP-RS and PSP-P, except for cognitive domains in life including any cognitive impairment (PSP mimic versus PSP-RS versus PSP-P: 23.5% versus 77.4% versus 68.6%; PSP mimic versus PSP-RS, *P < *0.01; PSP mimic versus PSP-P, *P < *0.05), frontal lobe dysfunction (PSP mimic versus PSP-RS; 23.5% versus 71.4%, *P < *0.01) and memory impairment (PSP mimic versus PSP-RS: 0% versus 45.7%, *P < *0.01). One PSP mimic had early apraxia of eyelid opening within 3 years of onset, while no cases of typical MSA, PSP-RS or PSP-P developed this feature (data not shown). In patients with apraxia of eyelid opening, some were initially labelled as having blepharospasm. However, their clinical label was changed to apraxia of eyelid opening due to the lack of, or partial, response to botulinum toxin in all injected patients.

In our previous study, we found that red flag features were useful in differentiating MSA from atypical PSP cases (MSA mimic), which masqueraded as MSA in life.^12^ Here, we examined the frequency of each red flag feature in the typical MSA, PSP mimic, PSP-RS and PSP-P groups (Table 4). Snoring was more common in typical MSA than PSP mimic and PSP-RS (typical MSA versus PSP mimic versus PSP-RS: 28.2% versus 0% versus 5.7%; typical MSA versus PSP mimic or PSP-RS, *P < *0.05). The cumulative frequency of red flag features during lifetime did not differ between PSP mimic and PSP-RS or PSP-P, except for cold hands and feet (PSP mimic versus PSP-P: 23.5% versus 0%, *P < *0.05).

**Table 4:** Red flag features in atypical MSA (PSP mimic) versus typical MSA or PSP

Pathological diagnosis	MSA		PSP	
	Typical MSA (*n *=* *177)	PSP mimic (*n *=* *17)	Typical PSP [PSP-RS (*n *=* *35)]	Typical PSP [PSP-P (*n *=* *35)]
**The frequency of red flag features during lifetime**				
1. Orofacial dystonia, % (*n*)	11.9 (21/177)	5.9 (1/17)	0 (0/35)	2.9 (1/35)
2. Inspiratory sighs, % (*n*)	20.3 (36/177)	11.8 (2/17)	5.7 (2/35)	2.9 (1/35)
3. Contractures of hands and feet, % (*n*)	9.0 (16/177)	11.8 (2/17)	5.7 (2/35)	8.6 (3/35)
4. Jerky myoclonic postural/action tremor, % (*n*)	23.7 (42/177)	29.4 (5/17)	5.7 (2/35)	8.6 (3/35)
5. Polyminimyoclonus, % (*n*)	14.7 (26/177)***	0 (0/17)	2.9 (1/35)	0 (0/35)
6. Severe dysphonia, % (*n*)	22.6 (40/177)	29.4 (5/17)	11.4 (4/35)	14.3 (5/35)
7. Severe dysarthria, % (*n*)	46.9 (83/177)***	23.5 (4/17)	48.6 (17/35)	11.4 (4/35)
8. Pathological laughter or crying, % (*n*)	23.2 (41/177)	29.4 (5/17)	22.9 (8/35)	17.1 (6/35)
9. Snoring, % (*n*)	28.2 (50/177)*^,^**	0 (0/17)	5.7 (2/35)	8.6 (3/35)
10. Disproportionate antecollis, % (*n*)	16.4 (29/177)	29.4 (5/17)	11.4 (4/35)	11.4 (4/35)
11. Camptocormia and/or Pisa syndrome, % (*n*)	2.8 (5/177)	11.8 (2/17)	0 (0/35)	0 (0/35)
12. Cold hands and feet, % (*n*)	20.3 (36/177)***	23.5 (4/17)^†^	2.9 (1/35)	0 (0/35)
**The number of red flag features**				
1. Cumulative frequency during lifetime				
Red flag score (the combination of red flags: 1, 2, 3, 4, 5, 6, 9), mean ± SD (OR, 95% CI)	1.3 ± 1.2 (3.0, 1.7–5.1)****	0.9 ± 0.9	0.4 ± 0.6	0.4 ± 0.7
2. Cumulative frequency within 10 years of onset				
Red flag score (the combination of red flags: 1, 2, 3, 4, 5, 6, 9), mean ± SD (OR, 95% CI)	1.2 ± 1.1 (3.0, 1.7–5.3)****	0.9 ± 0.9 (2.3, 1.1–4.8)^†^	0.3 ± 0.5	0.4 ± 0.7
3. Cumulative frequency within 3 years of onset				
Red flag score (1–12), mean ± SD (OR, 95% CI)	0.4 ± 0.8 (3.4, 1.1–10.3)***	0.4 ± 0.6	0.2 ± 0.4	0.1 ± 0.3

* *P *<* *0.05 typical MSA versus PSP mimic.

** *P *<* *0.05 typical MSA versus PSP-RS.

*** *P *<* *0.05 typical MSA versus PSP-P.

**** *P *<* *0.01 typical MSA versus PSP-P.

† *P *<* *0.05 PSP mimic versus PSP-P.

In our previous study, we identified seven red flag features that were helpful clinical pointers to differentiate MSA from atypical PSP (MSA mimic)^12^: orofacial dystonia, inspiratory sighs, contractures of hands and feet, jerky myoclonic postural/action tremor, polyminimyoclonus, severe dysphonia and snoring. Here, multiple logistic regression analysis was performed to explore whether the same combination may be used to discriminate PSP mimic from typical PSP. The PSP-P group was included as control because the majority of PSP mimics (63.6%, 7/11 PSP mimics who fulfilled the current diagnostic criteria for PSP) had an antemortem diagnosis of PSP-P. This analysis revealed an increased likelihood of having MSA than PSP-P when a patient developed any one of the seven red flag features within 10 years of disease onset (typical MSA versus PSP-P, OR: 3.0, 95% CI: 1.7–5.3, *P < *0.01; PSP mimic versus PSP-P, OR: 2.3, 95% CI: 1.1–4.8, *P < *0.05). The number of red flag features within 3 years of disease onset was also higher in typical MSA than PSP-P (OR: 3.4, 95% CI: 1.1–10.3, *P < *0.05), whereas no difference in the presence of red flag features was identified between the PSP mimic and PSP-P groups.

We then investigated the frequency of autonomic dysfunction between the typical MSA, PSP mimic, PSP-RS and PSP-P groups (Table 5). When compared with typical MSA, PSP-RS and PSP-P, more PSP mimics had early urinary incontinence within 3 years of onset (PSP mimic versus PSP-P: 23.5% versus 0%, *P < *0.05) than patients with PSP-P and had severe orthostatic hypotension than patients with PSP-RS (PSP mimic versus PSP-RS: 35.3% versus 0%, *P < *0.01). However, no statistical differences were seen in the other autonomic dysfunctions between PSP mimics and PSP-RS or PSP-P.

**Table 5:** Autonomic dysfunction in atypical MSA (PSP mimic) versus typical MSA or PSP

Pathological diagnosis	MSA		PSP	
	Typical MSA (*n *=* *177)	PSP mimic (*n *=* *17)	Typical PSP [PSP-RS (*n *=* *35)]	Typical PSP [PSP-P (*n *=* *35)]
**The frequency of autonomic dysfunction**				
Autonomic dysfunction (any including constipation), % (*n*)	100 (177/177)*^,^***	94.1 (16/17)	77.7 (27/35)	82.9 (29/35)
Urinary urgency, frequency, incomplete bladder emptying, or mild orthostatic hypotension, % (*n*)	76.3 (135/177)*	76.5 (13/17)	45.7 (16/35)	71.4 (25/35)
Early urinary urgency, frequency, incomplete bladder emptying, or mild orthostatic hypotension within 3 years of onset, % (*n*)	41.8 (74/177)*^,^**	17.6 (3/17)	11.4 (4/35)	14.3 (5/35)
Urinary incontinence, % (*n*)	76.3 (135/177)*^,^***	70.6 (12/17)	48.6 (17/35)	40 (14/35)
Early urinary incontinence within 3 years of onset, % (*n*)	31.1 (55/177)***	23.5 (4/17)^††^	11.4 (4/35)	0 (0/35)
Severe orthostatic hypotension, % (*n*)	58.8 (104/177)*^,^***	35.3 (6/17)^†^	0 (0/35)	5.7 (2/35)
Early severe orthostatic hypotension within 3 years of onset, % (*n*)	18.1 (32/177)**	11.8 (2/17)	2.9 (1/35)	0 (0/35)
**The number of autonomic dysfunction during lifetime**				
Severe orthostatic hypotension and/or urinary incontinence with use of urinary catheters, mean ± SD (OR, 95% CI)	1.2 ± 0.7 (18.9, 7.1–50.3)***	0.8 ± 0.6 (8.8, 2.8–27.8)^†††^	0.1 ± 0.4	0.1 ± 0.4
**The number of autonomic dysfunction within 3 years of onset**				
Urinary urgency, frequency, incontinence, incomplete bladder emptying, or mild or severe orthostatic hypotension, mean ± SD (OR, 95% CI)	1.1 ± 1.1 (4.2, 2.0–8.7)**	0.6 ± 0.9 (2.7, 1.1–6.5)^††^	0.3 ± 0.7	0.2 ± 0.6

* *P *<* *0.01 typical MSA versus PSP-RS.

** *P *<* *0.05 typical MSA versus PSP-P.

*** *P *<* *0.01 typical MSA versus PSP-P.

† *P *<* *0.01 PSP mimic versus PSP-RS.

†† *P *<* *0.05 PSP mimic versus PSP-P.

††† *P *<* *0.01 PSP mimic versus PSP-P.

We then carried out multiple logistic analysis and examined the likelihood of having MSA if a patient had the combination of severe autonomic dysfunction during their lifetime (severe orthostatic hypotension and/or urinary incontinence with use of urinary catheters) or autonomic dysfunction within 3 years of disease onset (urinary urgency, urinary frequency, incontinence, incomplete bladder emptying, or mild or severe orthostatic hypotension). When compared with patients with PSP-P, more patients with MSA (typical MSA and PSP mimics) had severe autonomic dysfunction than patients with PSP-P (typical MSA versus PSP-P, OR: 18.9, 95% CI: 7.1–50.3, *P < *0.01; PSP mimic versus PSP-P, OR: 8.8, 95% CI: 2.8–27.8, *P < *0.01). In addition, the number of autonomic dysfunction features within 3 years of disease onset was more frequent in MSA (typical MSA and PSP mimics) than PSP-P (typical MSA versus PSP-P, OR: 4.2, 95% CI: 2.0–8.7, *P < *0.01; PSP mimic versus PSP-P, OR: 2.7, 95% CI: 1.1–6.5, *P < *0.05).

**Figure 3:**
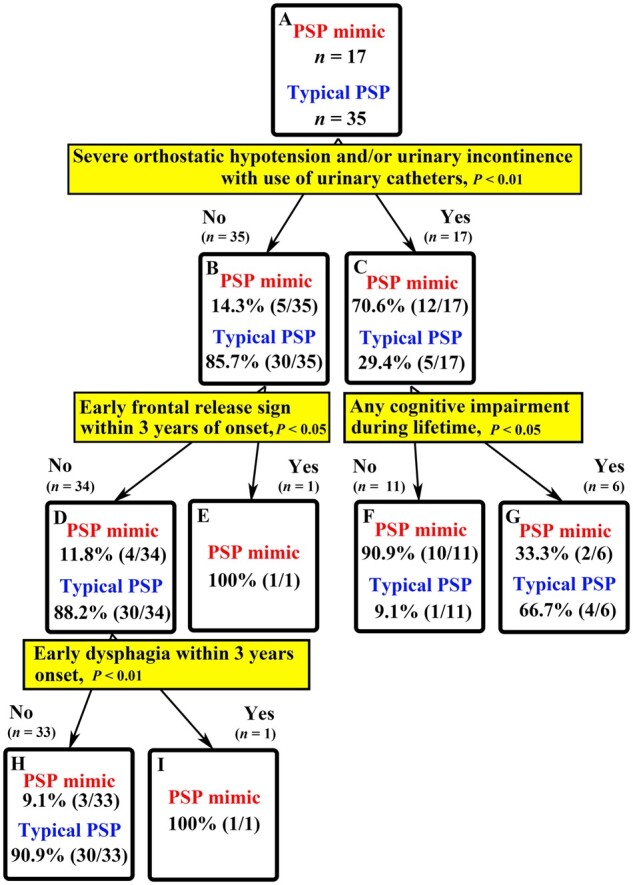
**Decision tree analysis: PSP mimic versus typical PSP (PSP-P).** Using all clinical features examined (62 items) outlined in Tables 4 and 5, and Supplementary Table 4, decision tree analysis was performed to distinguish PSP mimics from typical PSP (PSP-P). The decision tree algorithm automatically chooses the most determinant feature in the diagnostic process for the present cohort. It suggests which clinical feature should be heeded at the time of examination and/or in the future. Percentages indicate the probability of the underlying pathology [atypical MSA (PSP mimics) or typical PSP].

Finally, using all clinical features examined (62 items), decision tree analysis was performed to distinguish PSP mimics from patients with PSP-P (Fig. 3). These 62 items are outlined in Table 4 (15 items of red flag features), Table 5 (nine items of autonomic dysfunction) and Supplementary Table 4 (38 items of clinical features). In addition, with a view to identifying PSP mimics at the early stage of illness, we further performed decision tree analysis using 21 early clinical features within 3 years of onset (one item in Table 4; four items in Table 5; and 16 items in Supplementary Table 4) (Supplementary Fig. 3). Detailed descriptions of the decision tree analysis can be found in the Parkinson’s disease mimic section above.

## Discussion

Of patients with pathologically confirmed MSA, 81.2% (177/218) were correctly diagnosed as having MSA in life, whereas 18.8% (41/218) developed atypical clinical features and received a different antemortem diagnosis. Most atypical MSA cases masqueraded as either Parkinson’s disease (7.3%: 16/218) or PSP (7.8%: 17/218), and the majority of the Parkinson’s disease mimics (56.3%, 9/16) and PSP mimics (64.7%, 11/17) fulfilled current operational diagnostic criteria for Parkinson’s disease and PSP, respectively. These atypical cases of MSA can be considered as MSA/Parkinson’s disease or MSA/PSP ‘hybrids’ who showed characteristic clinical features of MSA as well as Parkinson’s disease or PSP. In the present study, 87.5% (14/16) of Parkinson’s disease mimics and all (17/17) PSP mimics were examined and followed up by experienced neurologists. However, we noticed that many clinicians were wavering between a diagnosis of MSA and Parkinson’s disease or PSP because of the presence of clinical features suggestive of both diseases. Despite the short disease course in Parkinson’s disease mimics, clinicians attached more importance to the presence of clinical features suggestive of Parkinson’s disease.

The present study has identified clinical pointers that help to identify MSA when clinicians are faced with ‘hybrid’ cases with mixed clinical features and waver between a diagnosis of MSA and Parkinson’s disease or PSP. These include the presence of (i) severe orthostatic hypotension and/or urinary incontinence with use of urinary catheter; (ii) urinary urgency, frequency, incontinence, incomplete bladder emptying, or mild or severe orthostatic hypotension within 3 years of onset; and (iii) selected red flag features, which help distinguish clinically atypical MSA (Parkinson’s disease or PSP mimics) from Parkinson’s disease or PSP cases, within 10 years of symptom onset. Several clinical red flag features were originally introduced to differentiate MSA from Parkinson’s disease.^13^ Five of the 15 original red flag features^13^—contractures of hands and feet, jerky myoclonic postural/action tremor, severe dysphonia/dysarthria, disproportionate antecollis, and cold hands and feet—are now incorporated into red flag features of the current diagnostic criteria.^8^ However, there remain unanswered questions as to whether such features can still be useful in identifying patients with MSA even if they develop atypical clinical symptoms suggestive of Parkinson’s disease or PSP. In the present study, we confirmed that the combination of seven selected red flag features were helpful in distinguishing clinically atypical MSA from typical Parkinson’s disease or PSP cases.

Decision tree analysis is a statistic model to predict the underlying pathology. These analyses in the present study show which clinical features should be heeded at the time of examination and/or in the future, especially when patients develop symptoms suggestive of both MSA and Parkinson’s disease or PSP. The features helpful to discriminate between Parkinson’s disease mimics and true Parkinson’s disease were: resting tremor; urinary incontinence; seven red flag features within 10 years of disease onset (orofacial dystonia, inspiratory sighs, contractures of hands and feet, polyminimyoclonus, severe dysarthria, pathological laughter or crying, and cold hands and feet); early rigidity within 3 years of onset; early falls within 3 years of disease onset; and falls within 10 years of onset (Fig. 2). Severe orthostatic hypotension and urinary incontinence with use of urinary catheter, early frontal release sign within 3 years of onset, any cognitive impairment, early dysphagia within 3 years of onset were useful in distinguishing PSP mimics from true PSP (Fig. 3). In addition, PSP mimics can be distinguished from typical PSP-P by examining the presence of urinary incontinence, dysphagia and/or ataxia within 3 years of onset (Supplementary Fig. 3). Validation by applying these features in a different cohort to determine their use to improve diagnostic accuracy is warranted.

The clinical pointers identified in the present study will allow physicians to predict the underlying pathology of MSA more accurately, especially when patients suspected of MSA exhibit overlapping clinical features that might suggest a diagnosis of Parkinson’s disease or PSP. It is noteworthy, however, that even with the application of these clinical pointers, 6.3% (1/16) of Parkinson’s disease mimics and 11.8% (2/17) of PSP mimics remained unidentifiable with high probability due to their paucity of core MSA features (Figs 2K and 3G).

Recurrent visual hallucinations are common in Parkinson’s disease, but are rare in MSA and PSP.^12^^,^^28^ Visual hallucinations were more frequently observed in the PSP mimics than in typical MSA (35.3% versus 5.1%). Visual hallucinations reported in these cases were mild and did not deteriorate over the disease course. No correlation was found between the presence of cognitive impairment and visual hallucination in PSP mimics or PSP cases. In some cases, hallucination occurred transiently in association with the use of medications and concurrent illnesses. It was neither vivid or in association with fluctuation in alertness and was therefore not consistent with the characteristics of visual hallucination in dementia with Lewy bodies. This explains why the presence of visual hallucination in these cases did not preclude clinicians from making the clinical diagnosis of PSP.

Contrary to other neurodegenerative diseases, the prevalence of concurrent amyloid-β, tau and Lewy body pathologies in MSA is not significantly higher than in age-matched healthy elderly individuals.^12^^,^^25^^,^^29^^,^^30^ In keeping with this, in the present study concomitant Lewy bodies were found in 7.3, 12.5 and 5.9% of typical MSA cases, Parkinson’s disease mimics and PSP mimics, respectively. Up to 23.7% of normal healthy individuals can have incidental Lewy bodies,^31–33^ and therefore, it is reasonable to consider concomitant Lewy bodies in all MSA subtypes in the present study as age-related. Consistent with previous reports,^25^^,^^29^ concomitant tau and amyloid-β deposition was minimal in our typical MSA cases (Supplementary Table 1). Concurrent tau and amyloid-β deposition occurred similarly in typical MSA cases and PSP mimics. While Parkinson’s disease mimics had more amyloid-β and less tau deposition than typical MSA cases (Table 1), we did not identify any difference in the prevalence of cognitive impairment when compared between typical MSA and Parkinson’s disease mimic. Moreover, none of the Parkinson’s disease mimics had intermediate or higher Alzheimer’s disease neuropathological change.^23^ Considering the minimal deposition of tau and amyloid-β in Parkinson’s disease mimics and PSP mimics, it is unlikely that these pathologies contributed to the atypical clinical presentation.

There are several limitations to the present study. First, in common with all brain bank-based retrospective clinicopathological studies, certain clinical features and clinical information including details of the levodopa response were not always recorded in every case and at every follow-up appointment. In particular, cognitive impairment in MSA may have been under-reported in some historical cases. Previous clinico-pathological studies have revealed that 20–37% of patients with MSA can develop cognitive impairment in life.^11^^,^^30^^,^^34^^-^^36^ Our data are comparable to those already reported in the literature; cognitive impairment was present in 20%, 31% and 23.5% of our typical MSA, Parkinson’s disease mimic and PSP mimic cases, respectively. Fewer Parkinson’s disease mimics than cases of typical MSA were reported to have developed RBD. It is not a common clinical practice in the UK to request polysomnography to confirm clinical suspicion of RBD. For this reason, most of our cases including Parkinson’s disease mimics, RBD was recorded as present based on behavioural descriptions by the bed partner. Where there was no documentation of polysomnography, we have presumed that these cases did not undergo it. The high percentage of PSP mimics with either vertical gaze palsy or slow vertical saccades was striking. Based on our data, we believe these clinical findings are truly pathological. The presence of these eye movement abnormalities was most likely the reason for these patients being diagnosed with PSP in life by their clinicians, most of whom were either experienced neurologists or movement disorders specialists. However, data on fast components optokinetic nystagmus or the effect of the doll’s head manoeuvre are missing in most PSP mimics. During data collection, we also avoided making assumptions concerning the presence of clinical features when they were not documented in the clinical records. Future prospective clinicopathological studies are required to address this specific research question. Second, it is possible that an inherent bias in history taking may have occurred in some cases. When clinicians suspect that a patient may have MSA, they are more likely to enquire about symptoms related to MSA, rather than other parkinsonian disorders. Third, the potential selection bias of brain bank post-mortem cases means that there is a higher prevalence of atypical cases compared to routine clinical practice. The QSBB specializes in parkinsonian disorders and it receives very few cases with sporadic late onset cerebellar ataxia.

In conclusion, with the largest sample size to date, we have elucidated clinical features of patients with atypical MSA that closely resemble Parkinson’s disease or PSP. In addition, we have identified clinical pointers to help distinguish these atypical MSA cases from patients with typical Parkinson’s disease or PSP. This is particularly useful in future clinical trials for Parkinson’s disease or PSP.

## Supplementary Material

awab017_Supplementary_Data

## Abbreviations

MSA: multiple system atrophy
OPCA: olivopontocerebellar atrophy
PSP-P/RS: progressive supranuclear palsy-parkinsonism/Richardson syndrome
SND: striatonigral degeneration
