# Supplementary material for: Identification of multiple system atrophy mimicking Parkinson’s disease or progressive supranuclear palsy
Source: Brain. 2021 Apr 5;144(4):1138–51. doi: 10.1093/brain/awab017 (PMC8310424; doi:10.1093/brain/awab017)
Supplement: awab017_Supplementary_Data [file awab017_supplementary_data.zip › brain-2020-01884-File010.pdf]

Supplementary material

Methods

Medical record review: clinical features

The clinical features that were investigated were: i) age of onset: age, in years, when any symptom considered to be attributable to the neurological disorder was first reported; ii) age at death; iii) time to final clinical diagnosis: time between the age of onset and the age when the last diagnosis recorded before death was mentioned; iv) disease duration to death: time between the age of onset and the age at death; v) latency between last examination and death: time from the last examination by specialists to death; vi) parkinsonism: recorded as positive if a patient developed tremor, bradykinesia, rigidity or positive pull test, vii) beneficial levodopa: moderate to good response described by a clinician, viii) resting tremor; ix) early resting tremor within three years of onset; x) postural/action tremor during lifetime; xi) early postural/action tremor within three years of onset; xii) intention tremor; xiii) early intension tremor within three years of onset; xiv) bradykinesia; xv) early bradykinesia within three years of onset; xvi) rigidity; xvii) early rigidity within three years of onset; xviii) positive pull test: recorded as positive if a patient was unable to maintain stability and would fall if not caught after a quick backwards pull; xix) early positive pull test within three years of onset; xx) falls: recorded as present if unprovoked falls were mentioned; xxi) early falls within three years of onset; xxii) freezing of gait ; xxiii) early

freezing of gait within three years of onset; xxiv) dysphagia within five years of onset; xxv) early dysphagia within three years of onset; xxvi) ataxia: recorded as present if gait ataxia, cerebellar dysarthria or limb ataxia was noted; xxvii) early ataxia within three years of onset; xxviii) stridor; xxix) early stridor within three years of onset; xxx) any cognitive impairment: recorded as positive if a patient developed memory impairment or frontal lobe dysfunction, the latter defined as presence of at least one of the following symptoms: personality change, executive dysfunction, disinhibition, or stereotypy; xxxi) early any cognitive impairment within three years of onset; xxxii) frontal lobe dysfunction; xxxiii) early frontal lobe dysfunction within three years of onset; xxxii) memory impairment; xxxiii) early memory impairment within three years of onset; xxxiv) frontal release signs: defined as presence of at least one of the following signs: Gegenhalten, snout reflex, palmomental reflex or grasp reflex; xxxv) early frontal release sign within three years of onset; xxxvi) vertical gaze palsy: recorded as present if upward or downward vertical gaze palsy was mentioned by a clinician; xxxvii) apraxia of eyelid opening; xxxviii) early apraxia of eyelid opening within three years of onset; xxxix) depression; xxxx) visual hallucination; xxxxi) early visual hallucination within three years of onset. This clinical feature is recorded as present when it is reported in the clinical record on at least one occasion. When clinical records did not describe a specific type of hallucination, we presumed that the patient's hallucinations were visual, because auditory hallucinations are very uncommon in this group of disorders.; xxxxii) REM sleep

behavior disorder (RBD): recorded as present if confirmed on polysomnography or if clinically suspected based on behavioral description by the bed partner; xxxxiii) features that raise the clinical suspicion of MSA. These comprise clinical features including orofacial dystonia, disproportionate antecollis, camptocormia and/or Pisa syndrome, contractures of hands or feet, inspiratory sighs, severe dysphonia, severe dysarthria, snoring, cold hands and feet, pathologic laughter and crying, jerky myoclonic postural/action tremor (Gilman *et al*., 2008; Köllensperger *et al*., 2008) and poly-mini-myoclonus (Okuma *et al*, 2005; Miki *et al*., 2019); xxxxiv) urinary urgency, frequency, incomplete bladder emptying, or mild orthostatic hypotension. Mild orthostatic hypotension was defined as a significant (20/10) drop that did not meet the above 30/15 drop; xxxxv) early urinary urgency, frequency, incomplete bladder emptying, or mild orthostatic hypotension within three years of onset; xxxxvi) urinary incontinence; xxxxvii) early urinary incontinence within three years of onset; xxxxviii) severe orthostatic hypotension; defined as a > 30 mm Hg systolic or 15 mm Hg diastolic blood pressure drop on standing, or repeated episodes of syncope; xxxxix) early severe orthostatic hypotension within three years of onset. In addition, because typical PD cases had a longer disease duration than MSA cases, we compared clinical features within the ten years of symptom onset between PD mimics and typical PD cases, which were significantly different in frequencies within three years of symptom onset or during life. The presence of early vertical gaze palsy, depression and RBD within three years of symptom

onset was unable to be examined due to difficulty in identifying their onsets in many cases.

References

Gilman S, Wenning GK, Low PA, Brooks DJ, Mathias CJ, Trojanowski JQ, et al. Second consensus statement on the diagnosis of multiple system atrophy. Neurology 2008; 71: 670-6.

Köllensperger M, Geser F, Seppi K, Stampfer-Kountchev M, Sawires M, Scherfler C, et al. Red flags for multiple system atrophy. Mov Disord 2008; 23: 1093-9.

Miki Y, Foti SC, Asi YT, Tsushima E, Quinn N, Ling H, et al. Improving diagnostic accuracy of multiple system atrophy: a clinicopathological study. Brain 2019; 142: 2813-2827

Okuma Y, Fujishima K, Miwa H, Mori H, Mizuno Y. Myoclonic tremulous movements in multiple system atrophy are a form of cortical myoclonus. Mov Disord 2005; 20: 451-6.
