# Supplementary material for: Identification of multiple system atrophy mimicking Parkinson’s disease or progressive supranuclear palsy
Source: Brain. 2021 Apr 5;144(4):1138–51. doi: 10.1093/brain/awab017 (PMC8310424; doi:10.1093/brain/awab017)
Supplement: awab017_Supplementary_Data [file awab017_supplementary_data.zip › brain-2020-01884-File011.pdf]

Supplementary table 1. Demographic data with statistical analysis between groups

Pathological diagnosis	MSA			PD	PSP		
	Typical MSA	PD mimic	PSP mimic	Typical PD	Typical PSP (PSP-RS)	Typical PSP (PSP-P)	*P* value (comparison between two groups)
**Clinical features**							
Number of patients (%)	177 (81.2)	16 (7.3)	17 (7.8)	35	35	35	
Male, % (N)	54.8 (97/177)	50 (8/16)	23.5 (4/17)	45.7 (16/35)	57.1 (20/35)	80 (28/35)	PSP mimic versus PSP-P, *P* <0.01
Age at onset (years), mean ± SD	56.0 ± 9.0	61.9 ± 10.7	61.7 ± 5.4	56.1 ± 11.7	66.1 ± 7.9	62.6 ± 10.4	Typical MSA versus PSP mimic, *P* <0.05; typical MSA versus PSP-RS, *P* <0.01; typical MSA versus PSP-P, *P* <0.05
Age at death (years), mean ± SD	63.6 ± 8.2	68.7 ± 9.5	67.5 ± 5.4	78.2 ± 8.1	74.3 ± 7.2	73.1 ± 10.7	Typical MSA versus typical PD, *P* <0.01; typical MSA verus PSP-RS, *P* <0.01; typical MSA versus PSP-P, *P* <0.01; PD mimic versus typical PD, *P* <0.05; PSP mimic versus PSP-RS, *P* <0.05
Time to final diagnosis (years), mean ± SD	4.2 ± 2.5	1.3 ± 1.5	3.9 ± 1.7	1.7 ± 1.8	3.6 ± 2.2	6.2 ± 2.6	Typical MSA versus typical PD, *P* <0.01; typical MSA versus PD mimic, *P* <0.01; typical MSA versus PSP-P, *P* <0.01; PD mimic versus PSP mimic, *P* <0.01; PSP mimic versus PSP-P, *P* <0.05
Disease duration to death (years), mean ± SD	7.6 ± 3.0	7.1 ± 4.1	6.4 ± 1.8	21.9 ± 6.6	8.0 ± 3.0	10.5 ± 4.0	Typical MSA versus typical PD, *P* <0.01; typical MSA versus PSP-P, *P* <0.01; PD mimic versus typical PD, *P* <0.01; PSP mimic versus PSP-P, *P* <0.01
Latency between last examination and death (years), mean ± SD	1.1 ± 1.1	0.8 ± 0.9	0.8 ± 0.9	2.1 ± 2.7	1.2 ± 1.6	1.4 ± 1.7	N.S.
MSA clinical subtypes							
MSA-P, % (N)	62.1 (110/177)	-	-	-	-	-	N.A
MSA-C, % (N)	37.9 (67/177)	-	-	-	-	-	N.A
**Pathological features**							
MSA pathological subtype							
MSA-SND, % (N)	29.4 (52/177)	50 (8/16)	70.6 (12/17)	-	-	-	Typical MSA versus PSP mimic, *P* <0.01
MSA-OPCA, % (N)	34.5 (61/177)	18.8 (3/16)	0 (0/17)	-	-	-	Typical MSA versus PSP mimic, *P* <0.05
MSA-mixed, % (N)	33.9 (61/177)	31.3 (5/16)	29.4 (5/17)	-	-	-	N.S.
Minimal change, % (N)	1.7 (3/177)	0 (0/16)	0 (0/17)	-	-	-	N.S.
CERAD plaque score, median (25th, 75th percentile)	0 (0, 0)	0 (0, 3)	0 (0, 1)	0 (0, 1)	0 (0, 1)	0 (0,1)	Typical MSA versus PD mimic, *P* <0.01
NFT stage, median (25th, 75th percentile)	I (0, I)	0 (0, 0)	I (0.5, I)	II (I, II)	II (0, II)	II (0, II)	Typical MSA versus PD mimic, *P* <0.05; typical MSA versus typical PD, *P* <0.01; typical MSA versus PSP-P, *P* <0.01; PD mimic versus typical PD, *P* <0.01
Lewy body pathology, % (N)	7.3 (13/177)	12.5 (2/16)	5.9 (1/17)	100 (35/35)	22.9 (8/35)	17.1 (6/35)	N.S.
Brainstem-predominant, % (N)	-	-	-	0 (0/35)	-	-	N.A.
Limbic (transitional), % (N)	-	-	-	14.3 (5/35)	-	-	N.A.
Diffuse neocortical, % (N)	-	-	-	85.7 (30/35)	-	-	N.A.

Abbreviations: NS: not significant; NA: not available.

Supplementary table 2. Key clinical features of PD or PSP mimics

Cases	Final clinical diagnosis	Pathological diagnosis	Retrospective application of the current diagnostic criteria for PD or PSP	Age at death	Disease duration	Parkinsonism	Levodopa response	Resting tremor	Postural/Action tremor	Positive pull test	Early Falls	Ataxia	Stridor	Vertical gaze palsy	Apraxia of eyelid opening	Hallucination	Autonomic dysfunction*	Urinary incontinence	Severe Orthostatic hypotension
1	PD	MSA		74	4	+													
2	PD	MSA		57	3	+	+							+			+	+	+
3	PD	MSA	Clinically probable PD	66	4	+	+									+	+		
4	PD	MSA	Clinically probable PD	67	18	+	+			+					+		+	+	
5	PD	MSA	Clinically established PD	63	Unknown	+	+			+								+	
6	PD	MSA	Clinically probable PD	70	9	+	+	+						+			+		
7	PD	MSA	Clinically probable PD	51	3	+	+										+	+	
8	PD	MSA		80	2	+	+	+			+						+	+	
9	PD	MSA		72	5	+				+	+			+		+	+		
10	PD	MSA		64	8	+		+						+			+	+	+
11	PD	MSA	Clinically probable PD	75	9	+	+	+								+	+	+	
12	PD	MSA	Clinically probable PD	77	9	+	+										+	+	
13	PD	MSA		76	6	+	+										+	+	+
14	PD	MSA	Clinically probable PD	51	11	+	+			+							+	+	
15	PD	MSA		81	6	+	+				+							+	
16	PD	MSA	Clinically probable PD	75	10	+	+									+			
17	PSP	MSA		73	7	+		+			+	+		+					+
18	PSP	MSA	Suggestive PSP-P	65	8	+		+		+				+			+		
19	PSP	MSA		72	8	+		+		+		+		+		+	+	+	+
20	PSP	MSA		61	4	+			+	+	+			+	+		+	+	+
21	PSP	MSA	Probable PSP-RS	63	9	+	+		+	+	+			+	+	+	+	+	
22	PSP	MSA	Probable PSP-P	64	7	+				+				+			+	+	
23	PSP	MSA	Suggestive PSP-P	70	7	+				+				+			+	+	
24	PSP	MSA	Possible PSP with progressive gait freezing	65	3	+					+					+	+	+	
25	PSP	MSA	Possible PSP with progressive gait freezing	74	6	+				+	+				+	+	+	+	
26	PSP	MSA		78	3	+		+	+		+			+					+
27	PSP	MSA	Probable PSP-P	62	7	+		+		+	+			+			+	+	
28	PSP	MSA		67	8	+	+			+	+			+	+		+	+	+
29	PSP	MSA	Probable PSP-P	68	6	+		+	+	+				+		+	+		
30	PSP	MSA	Probable PSP-RS	66	6	+					+					+			
31	PSP	MSA	Suggestive PSP-P	74	8	+	+										+	+	
32	PSP	MSA		67	6	+			+	+	+	+		+				+	+
33	PSP	MSA	Probable PSP-P	58	5	+	+	+	+					+	+		+	+	

* Urinary urgency, frequency, incomplete bladder emptying, or mild orthostatic hypotension

Supplementary table 3. Clinical features in atypical MSA (PD mimic) versus typical MSA or PD

Pathological diagnosis	MSA		PD
**The frequency of clinical features**	**Typical MSA (n = 177)**	**PD mimic (n = 16)**	**Typical PD (n = 35)**
Parkinsonism during lifetime (tremor, bradykinesia, rigidity or positive pull test)	94.4 (167/177)	100 (16/16)	100 (35/35)
Levodopa response (moderate to good), % (n)	41.1 (53/129)**,***	81.3 (13/16)	100 (35/35)
Tremor			
Resting tremor, % (n)	26.6 (47/177)***	25 (4/16)††	91.4 (32/35)
Early resting tremor within 3 years of onset, % (n)	9.6 (17/177)***	12.5 (2/16)	40 (14/35)
Postural/action tremor, % (n)	33.3 (59/177)*	0 (0/16)	22.9 (8/35)
Early postural/action tremor within 3 years of onset, % (n)	10.7 (19/177)	0 (0/16)	11.4 (4/35)
Intention tremor, % (n)	17.5 (31/177)	0 (0/16)	5.7 (2/35)
Early Intention tremor within 3 years of onset, % (n)	3.4 (6/177)	0 (0/16)	0 (0/35)
Bradykinesia, % (n)	79.1 (140/177)***	100 (16/16)	100 (35/35)
Early bradykinesia within 3 years of onset, % (n)	46.3 (82/177)	62.5 (10/16)	62.8 (22/35)
Rigidity, % (n)	78.0 (138/177)***	100 (16/16)	100 (35/35)
Early rigidity within 3 years of onset, % (n)	32.8 (58/177)*,***	68.8 (11/16)	62.9 (22/35)
Positive pull test, % (n)	32.2 (57/177)	25 (4/16)	20 (7/35)
Early positive pull test within 3 years of onset, % (n)	10.2 (18/177)	0 (0/16)	0 (0/35)
Falls, % (n)	83.1 (147/177)	93.8 (15/16)	88.6 (31/35)
Early falls within 3 years of onset, % (n)	38.4 (68/177)***	18.8 (3/16)	5.7 (2/35)
Freezing of gait, % (n)	16.9 (30/177)***	25 (4/16)†	68.6 (24/35)
Early freezing of gait within 3 years of onset, % (n)	5.1 (9/177)	6.3 (1/16)	0 (0/35)
Dysphagia within 5 years of onset, % (n)	37.9 (67/177)***	31.3 (5/16)	11.4 (4/35)
Early dysphagia within 3 years of onset, % (n)	6.8 (12/177)	12.5 (2/16)	2.9 (1/35)
Ataxia, % (n)	64.4 (114/177)**,***	0 (0/16)	0 (0/35)
Early ataxia within 3 years of onset, % (n)	33.9 (60/177)*,***	0 (0/16)	0 (0/35)
Stridor, % (n)	31.1 (55/177)*,***	0 (0/16)	0 (0/35)
Early stridor within 3 years of onset, % (n)	1.1 (2/177)	0 (0/16)	0 (0/35)
Any cognitive impairment (Impairment of frontal lobe function or memory), % (n)	19.8 (35/177)***	31.3 (5/16)	42.3 (15/35)
Early cognitive impairment (impairment of frontal lobe function or memory) within 3 years of onset, % (n)	6.2 (11/177)	6.3 (1/16)	0 (0/35)
Frontal lobe dysfunction, % (n)	12.4 (22/177)	6.3 (1/16)	22.9 (8/35)
Early frontal lobe dysfunction within 3 years of onset, % (n)	3.4 (6/177)	0 (0/16)	0 (0/35)
Memory impairment during lifetime, % (n)	10.2 (18/177)***	25 (4/16)	31.4 (11/35)
Early memory impairment within 3 years of onset, % (n)	4.0 (7/177)	6.3 (1/16)	0 (0/35)
Frontal release sign, % (n)	11.9 (21/177)	6.3 (1/16)	2.9 (1/35)
Early frontal release sign within 3 years of onset, % (n)	1.7 (3/177)	0 (0/16)	0 (0/35)
Vertical gaze palsy, % (n)	20.3 (36/177)	25 (4/16)	11.4 (4/35)
Apraxia of eyelid opening, % (n)	4.0 (7/177)	6.3 (1/16)	2.9 (1/35)
Depression, % (n)	42.9 (76/177)	37.5 (6/16)	34.3 (12/35)
Visual hallucination, % (n)	5.1 (9/177)*,***	25 (4/16)††	80 (28/35)
Early visual hallucination within 3 years of onset, % (n)	1.1 (2/177)	0 (0/16)	0 (0/35)
REM sleep behaviour disorder, % (n)	41.8 (74/177)*	6.3 (1/16)	40 (14/35)

**P* <0.05 typical MSA vs PD mimic; ***P* <0.01 typical MSA vs PD mimic; ****P* <0.01 typical MSA vs typical PD; †*P* <0.05 PD mimic vs typcial PD; ††*P* <0.01 PD mimic vs typical PD

Supplementary table 4. Clinical features in atypical MSA (PSP mimic) versus typical MSA or PSP

Pathological diagnosis	MSA		PSP	
**The frequency of clinical features**	**Typical MSA (n = 177)**	**PSP mimic (n = 17)**	**Typical PSP (PSP-RS (n = 35))**	**Typical PSP (PSP-P (n = 35))**
Parkinsonism (tremor, bradykinesia, rigidity or positive pull test)	94.4 (167/177)	100 (17/17)	94.3 (33/35)	100 (35/35)
Levodopa response (moderate to good), % (n)	41.1 (53/129)	33.3 (4/12)	15.4 (4/26)	31.6 (6/19)
Tremor				
Resting tremor, % (n)	26.6 (47/177)	41.2 (7/17)	14.3 (5/35)	40 (14/35)
Early resting tremor within 3 years of onset, % (n)	9.6 (17/177)	17.6 (3/17)	2.9 (1/35)	25.7 (9/35)
Postural/action tremor, % (n)	33.3 (59/177)	35.3 (6/17)	14.3 (5/35)	22.9 (8/35)
Early postural/action tremor within 3 years of onset, % (n)	10.7 (19/177)	23.5 (4/17)	5.7 (2/35)	17.1 (6/35)
Intention tremor, % (n)	17.5 (31/177)	11.8 (2/17)	2.9 (1/35)	2.9 (1/35)
Early Intention tremor within 3 years of onset, % (n)	3.4 (6/177)	0 (0/17)	0 (0/35)	0 (0/35)
Bradykinesia, % (n)	79.1 (140/177)	94.1 (16/17)	88.6 (31/35)	94.2 (33/35)
Early bradykinesia within 3 years of onset, % (n)	46.3 (82/177)*,††	82.4 (14/17)	60 (21/35)	77.1 (27/35)
Rigidity, % (n)	78.0 (138/177)	88.2 (15/17)	82.9 (29/35)	88.6 (31/35)
Early rigidity within 3 years of onset, % (n)	32.8 (58/177)	58.8 (10/17)	40 (14/35)	48.6 (17/35)
Positive pull test, % (n)	32.2 (57/177)*,****	64.7 (11/17)	60 (21/35)	51.4 (18/35)
Early positive pull test within 3 years of onset, % (n)	10.2 (18/177)	29.4 (5/17)	20 (7/35)	8.6 (3/35)
Falls, % (n)	83.1 (147/177)***	100 (17/17)	100 (35/35)	97.1 (34/35)
Early falls within 3 years of onset, % (n)	38.4 (68/177)****	58.8 (10/17)	88.6 (31/35)	28.6 (10/35)
Freezing of gait, % (n)	16.9 (30/177)†	29.4 (5/17)	20 (7/35)	40 (14/35)
Early freezing of gait within 3 years of onset, % (n)	2.8 (5/177)	0 (0/17)	8.6 (3/35)	5.7 (2/35)
Dysphagia within 5 years of onset, % (n)	37.9 (67/177)	58.8 (10/17)	57.1 (20/35)	25.7 (9/35)
Early dysphagia within 3 years of onset, % (n)	6.8 (12/177)	17.6 (3/17)	14.3 (5/35)	0 (0/35)
Ataxia, % (n)	64.4 (114/177)**,****,††	17.6 (3/17)	22.9 (8/35)	8.6 (3/35)
Early ataxia within 3 years of onset, % (n)	33.9 (60/177)****,††	11.8 (2/17)	5.7 (2/35)	0 (0/35)
Stridor, % (n)	31.1 (55/177)*,****,††	0 (0/17)	0 (0/35)	0 (0/35)
Early stridor within 3 years of onset, % (n)	1.1 (2/177)	0 (0/17)	0 (0/35)	0 (0/35)
Any cognitive impairment (Impairment of frontal lobe function or memory), % (n)	19.8 (35/177)****,††	23.5 (4/17)‡, ‡‡	77.4 (27/35)	68.6 (24/35)
Early cognitive impairment (impairment of frontal lobe function or memory) within 3 years of onset, % (n)	6.2 (11/177)****	5.9 (1/17)	31.4 (11/35)	20 (7/35)
Frontal lobe dysfunction, % (n)	12.4 (22/177)****,††	23.5 (4/17)‡	71.4 (25/35)	57.1 (20/35)
Early Frontal lobe dysfunction within 3 years of onset, % (n)	3.4 (6/177)****,††	5.9 (1/17)	22.9 (8/35)	20 (7/35)
Memory impairment, % (n)	10.2 (18/177)****	0 (0/17)‡	45.7 (16/35)	25.7 (9/35)
Early memory impairment within 3 years of onset, % (n)	4.0 (7/177)***	0 (0/17)	20 (7/35)	5.7 (2/35)
Frontal release sign, % (n)	11.9 (21/177)	17.6 (3/17)	28.6 (10/35)	25.7 (9/35)
Early frontal release sign within 3 years of onset, % (n)	1.7 (3/177)	11.8 (2/17)	2.9 (1/35)	0 (0/35)
Vertical gaze palsy, % (n)	20.3 (36/177)**,****,††	76.5 (13/17)	97.1 (34/35)	82.9 (29/35)
Apraxia of eyelid opening, % (n)	4.0 (7/177)**,****,††	29.4 (5/17)	37.1 (13/35)	40 (14/35)
Depression, % (n)	42.9 (76/177)	35.3 (6/17)	34.3 (12/35)	40 (14/35)
Visual hallucination, % (n)	5.1 (9/177)**	35.3 (6/17)	11.4 (4/35)	17.1 (6/35)
Early visual hallucination within 3 years of onset, % (n)	1.1 (2/177)	0 (0/17)	5.7 (2/35)	0 (0/35)
REM sleep behaviour disorder, % (n)	41.8 (74/177)****,†	11.8 (2/17)	5.7 (2/35)	14.3 (5/35)

**P* <0.05 typical MSA vs PSP mimic; ***P* <0.01 typical MSA vs PSP mimic; ****P* <0.05 typical MSA vs PSP-RS; *****P* <0.01 typical MSA vs PSP-RS; †*P* <0.05 typical MSA vs PSP-P; ††*P* <0.01 typical MSA vs PSP-P; ‡*P* <0.01 PSP mimic vs PSP-RS; ‡‡ *P* <0.05 PSP mimic vs PSP-P
