# Supplementary material for: Identification of multiple system atrophy mimicking Parkinson’s disease or progressive supranuclear palsy
Source: Brain. 2021 Apr 5;144(4):1138–51. doi: 10.1093/brain/awab017 (PMC8310424; doi:10.1093/brain/awab017)

Supplementary Fig. 1 Frequencies of clinical features over time in typical MSA versus PD mimic versus typical PD.

Clinical features within three and ten years of onset, and during lifetime are shown.

Supplmentary Fig. 1

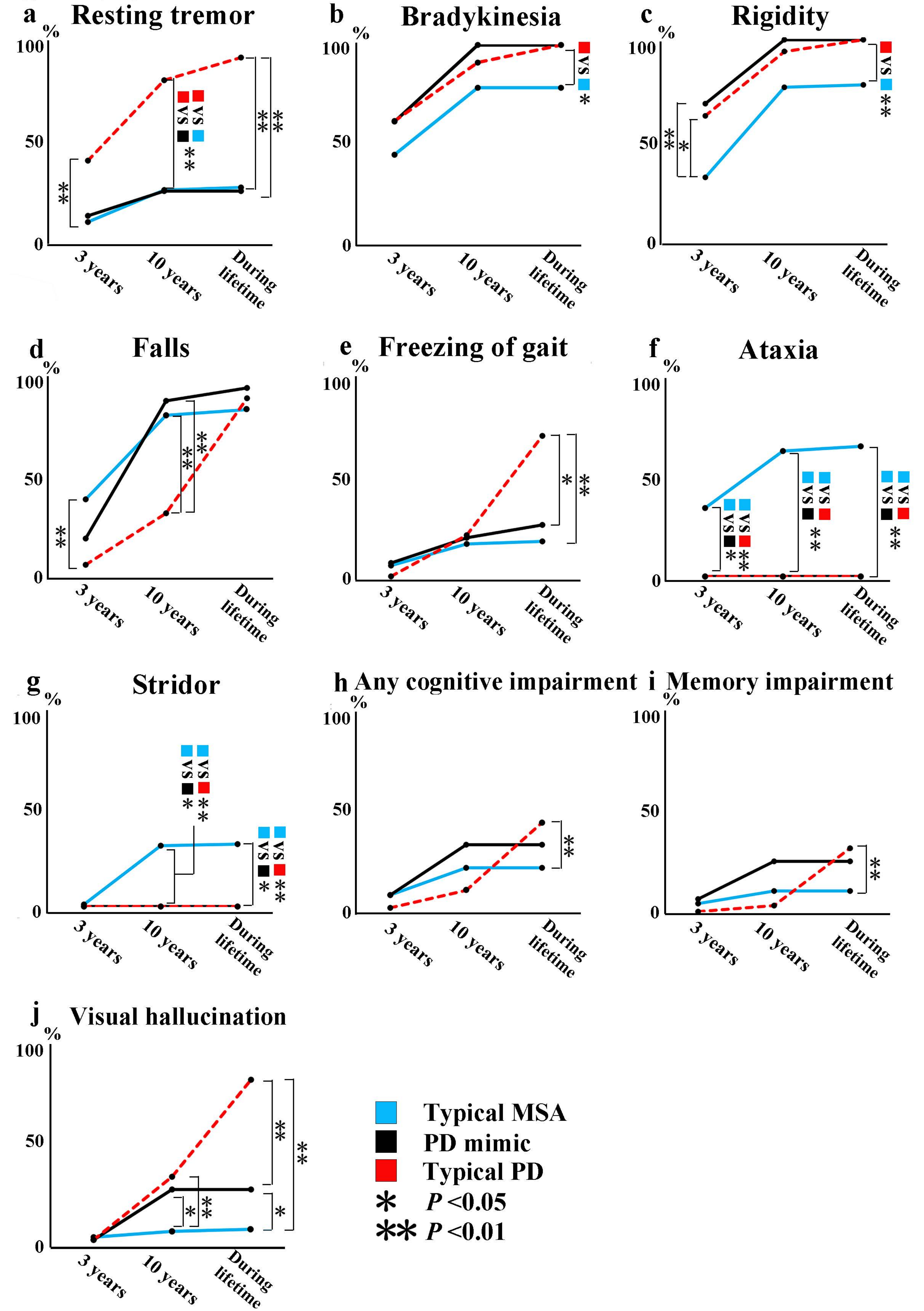

Supplementary Fig. 2 Frequencies of autonomic dysfunction over time in typical MSA versus PD mimic versus typical PD

Autonomic dysfunctions within three and ten years of onset, and during lifetime are shown.

Supplementary Fig. 2

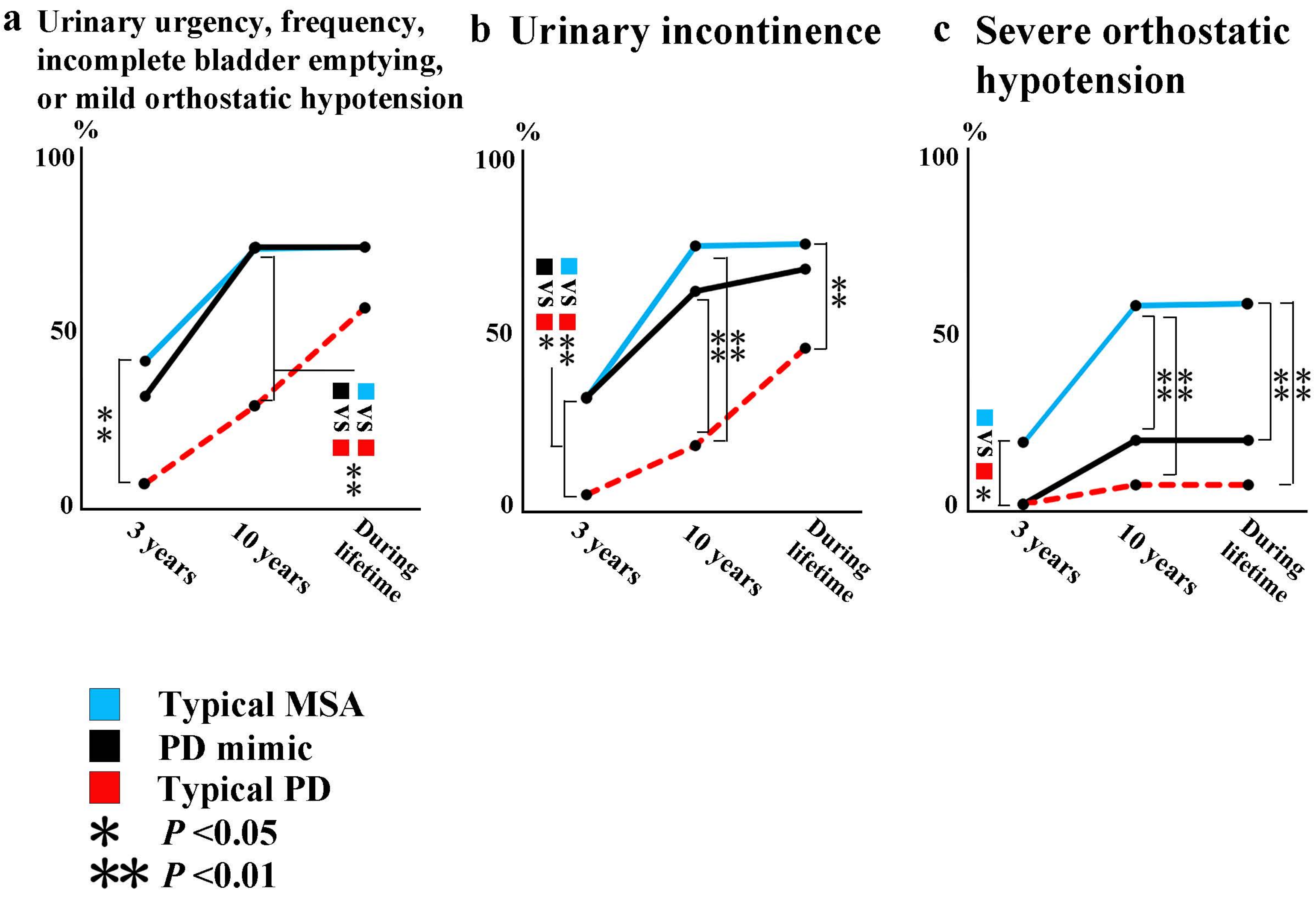

Supplementary Fig. 3 Decision tree analysis using 21 early clinical features within three years of symptom onset: PSP mimic versus typical PSP (PSP-P)

Using 21 early clinical features within three years of onset, outlined in tables 4, 5 and supplementary table 4, decision tree analysis was performed to distinguish PSP mimics from typical PSP (PSP-P). The decision tree algorithm automatically chooses the most determinant feature in the diagnostic process for the present cohort. Percentages indicate possible diagnostic accuracy of atypical MSA (PSP mimics) or typical PSP.

Supplementary Fig. 3

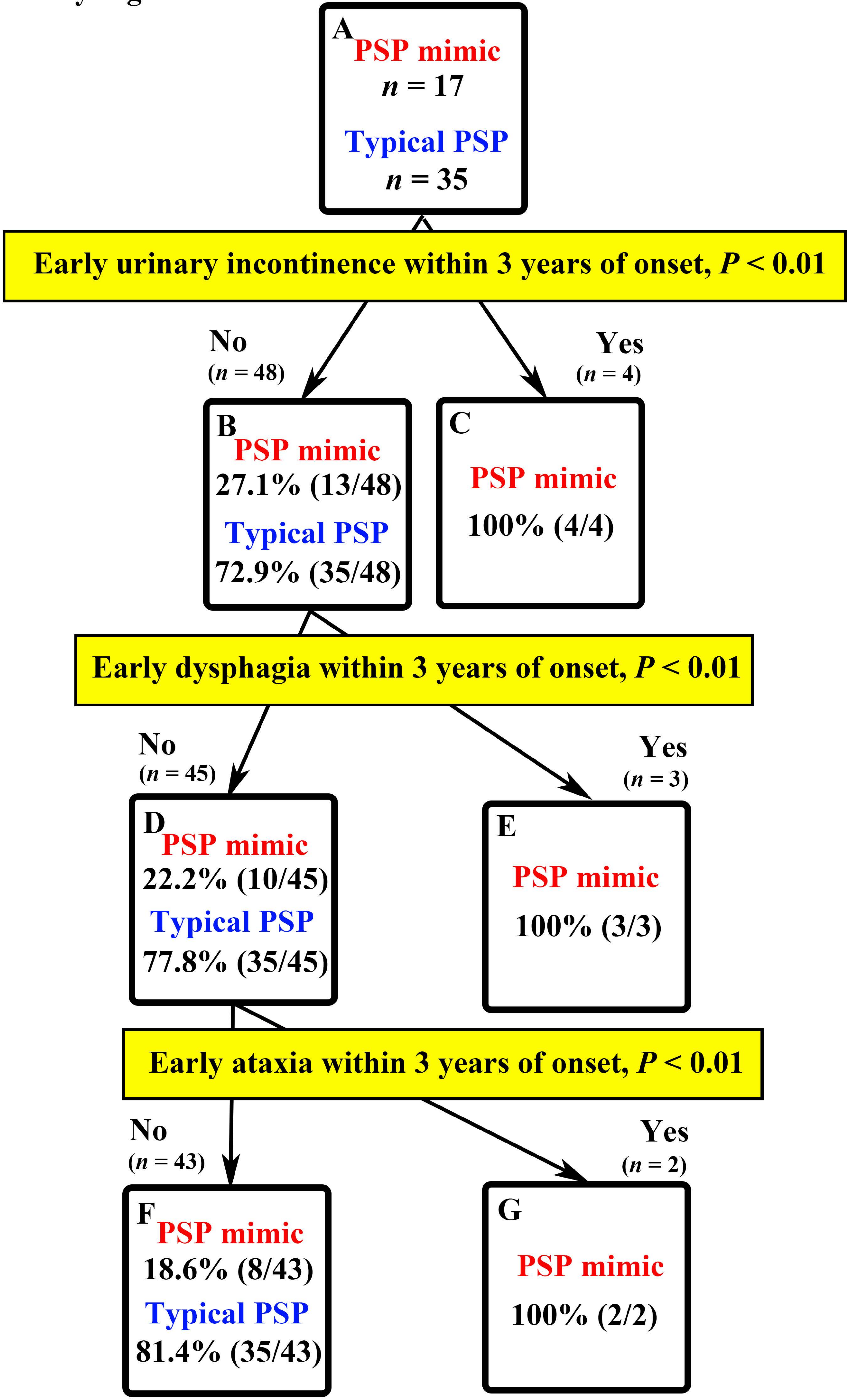

Supplement: awab017_Supplementary_Data [file awab017_supplementary_data.zip › brain-2020-01884-File012.pdf]
